# A Deep Learning Framework for Driving Behavior Identification on In-Vehicle CAN-BUS Sensor Data

**DOI:** 10.3390/s19061356

**Published:** 2019-03-18

**Authors:** Jun Zhang, ZhongCheng Wu, Fang Li, Chengjun Xie, Tingting Ren, Jie Chen, Liu Liu

**Affiliations:** 1 High Magnetic Field Laboratory, and Hefei Institutes of Physical Science, Chinese Academy of Sciences, Hefei 230031, China; zcwu@iim.ac.cn (Z.C.W.); lif@hmfl.ac.cn (F.L.); ttren@hmfl.ac.cn (T.R.); cj2016@mail.ustc.edu.cn (J.C.); 2 University of Science and Technology of China, Hefei 230026, China; 3 Institute of Intelligent Machines, and Hefei Institute of Physical Science, Chinese Academy of Sciences, Hefei 230031, China; liuliu66@mail.ustc.edu.cn

**Keywords:** driving behavior identification, deep learning, attention mechanism, CNN, LSTM, GRU

## Abstract

Human driving behaviors are personalized and unique, and the automobile fingerprint of drivers could be helpful to automatically identify different driving behaviors and further be applied in fields such as auto-theft systems. Current research suggests that in-vehicle Controller Area Network-BUS (CAN-BUS) data can be used as an effective representation of driving behavior for recognizing different drivers. However, it is difficult to capture complex temporal features of driving behaviors in traditional methods. This paper proposes an end-to-end deep learning framework by fusing convolutional neural networks and recurrent neural networks with an attention mechanism, which is more suitable for time series CAN-BUS sensor data. The proposed method can automatically learn features of driving behaviors and model temporal features without professional knowledge in features modeling. Moreover, the method can capture salient structure features of high-dimensional sensor data and explore the correlations among multi-sensor data for rich feature representations of driving behaviors. Experimental results show that the proposed framework performs well in the real world driving behavior identification task, outperforming the state-of-the-art methods.

## 1. Introduction

Everyone has unique driving habits such as fixed speed, acceleration and braking habits, which could be considered as a fingerprint [1]. Thus, drivers’ characteristics under driving conditions could be extracted through the analysis of driving behaviors. Considering different sources of data, we classify most current driving behavior identification models into three classes, that is, visual image or video-based, simulation data-based [2,3,4] and CAN-BUS(Controller Area Network-BUS)/smartphone multi-sensors data-based [5]. Among these, the visual data can be viewed as a special case of “multi-sensors data”, and the third one, which is more effective and favorable, is our focus in this paper. Specifically, we neglect analyzing visual data due to the poor amount of training data.

Generally, multi-sensors data are made up of in-vehicle’s CAN data and Smartphone data. The in-vehicle’s CAN data include the steering wheel, vehicle speed, engine speed, brake position, etc., while the smartphone data include speed, orientation, three-axis accelerometer, etc. Several works proposed driver identification methods based on in-vehicle’s CAN-BUS data [1,6,7,8]. In [9,10], deep sparse autoencoder (DSAE) was developed to extract hidden features for visualization of driving behavior, which was helpful to recognize distinctive driving behavior patterns in continuous data. Some researchers adopted the three-axis accelerometer of an Android-based smart phone to record and analyze various driver behaviors, external road conditions [11], the degree of aggressiveness of each driver [12], and accident detection [13]. From the above works, it was concluded that driving pattern analysis is an efficient method for driver identification.

With the rapid development of Internet of Vehicles (IoV) technology and the popularization of smart terminal devices like car onboard diagnostic (OBD) devices, multi-dimensional CAN-BUS data can be easily captured for driving behavior recognition and vehicle owner identification. Driving behavior identification is essentially a classification task based on in-vehicle’s CAN-BUS data. It is important to choose key features from these driving data, and find the combination of features. For instance, driver A likes to accelerate quickly at startup while driver B is used to driving at a slow speed. However, previous works developed complex feature selection techniques to improve the performance of driving behavior identification. Among them, there exist several difficulties in manual feature combination. The first one is feature explosion difficulty, which is hard for experts to explore exhaustively, especially when the number of raw features is huge. The second one is that features are difficult to design, where part of the available training data has been desensitized due to individual privacy protection, leading to impossibility in simply performing feature engineering based on common sense. Third, combined features are difficult to identify and recognize, since generally most feature interactions are hidden behind numerous data and difficult to mine, which can only be captured automatically by machine learning. Fourth, the temporal dynamics of feature activations is difficult to model explicitly. Moreover, the issue of how to effectively train the model is also a challenge, since CAN-BUS data sometimes are massive and high-dimensional, therefore large feature space will lead to a growth of parameter number, increasing the complexity of model training.

Generally, the activity recognition or identification of drivers relies on the combinations of different CAN-BUS sensor data. However, traditional feature extraction methods for driving behavior identification adopt sliding window for static and periodic activities modeling [14]. In driving behavior identification, complex high level behaviors (e.g., trip-long, day-long or more) are usually scaled up since engineering features are not related to “units of driving behaviors” but to the results of complex sequences of motor movements. However, in CAN-BUS data, multiple sensors yield multivariate time series, for instance, a single 3-axis accelerometer produces a 3-dimensional time series. Thus, it is desirable to consider the spatial dependency among multiple sensors or across the axes of accelerometers and gyroscopes, as well as the dependency along the temporal dimension.

**Figure 1:**
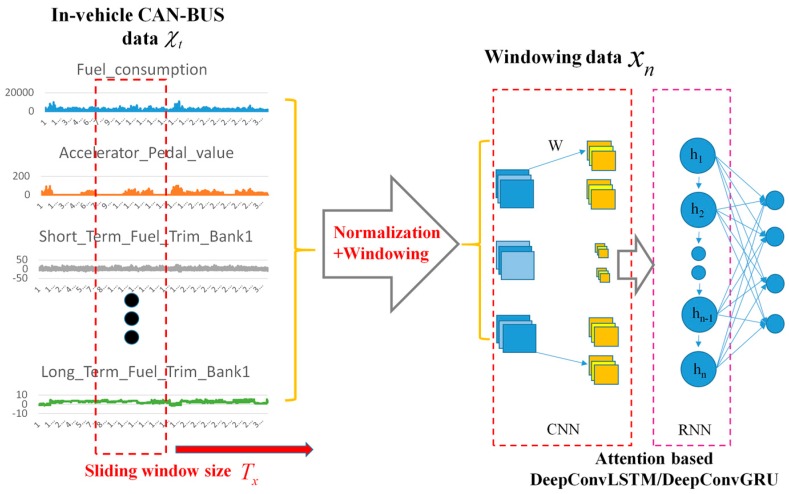
The outline of our approach.

In this paper, we propose a deep learning framework by fusing deep convolutional and recurrent neural network, denoted as attention-based DeepConvGRU and DeepConvLSTM respectively, for driving behavior identification. The outline of our approach is illustrated in Figure 1. First, in-vehicle CAN-BUS sensor data are collected for each driver to characterize the drivers’ driving behaviors. Second, the time series CAN-BUS data are split into fragments by normalization and sliding window. Finally, the annotated data are fed into our proposed deep learning framework for driver behavior identification.

The main contributions are summarized as follows:

Our framework can perform automatic activity recognition on real-time multi-dimensional in-vehicle CAN-BUS sensor data, capturing local dependency among the data in temporal dimension as well as across spatial locations.

By introducing the attention mechanism, our model can capture salient structures of high-dimensional sensor data and explore the correlations among multi-channel sensor data for rich feature representations, improving the learning performance of the model.

Our framework can perform end-to-end training without any feature selection and work directly on the raw sensor data with simple pre-processing, making it universally applicable.

## 2. Materials and Methods

### 2.1. Related Works

Many state-of-the-art models were used in modeling individual driving behaviors, such as Gaussian Mixture Model (GMM) [2,6,15,16], Hidden Markov Model (HMM) [4,6,17], K-means [8], Support Vector Machine (SVM), Random Forest, Naive Bayes (NB), K-Nearest Neighbor (KNN) [1,8], Multilayer Perceptron (MLP), Fuzzy-Neural-Network (FNN), statistical method [3], Decision Tree (DT) and Symbolic Aggregate Approximation (SAX). However, most of them had various shortcomings. HMM was limited to contextual information representation, based on the hypothesis that the output observations were strictly independent and the current state was only related to the previous state (first-order Markov model). In addition, KNN was affected by unbalanced training data, which resulted in higher time complexity when calculating the distance from the unknown sample to all known samples. Moreover, the model of NB was based on the hypothesis that sample attributes were independent from each other. Therefore, NB might yield a lower classification performance when the number of sample attributes or the correlation between attributes became larger, which required enough samples to calculate the overall distribution of each class and the probability distribution of each sample. For the DT model, it had to scan and sort the data set repeatedly during model construction, which would increase the complexity and reduce the classification accuracy.

Deep learning has a great advantage in feature learning. For example, Convolutional Neural Network (CNN) [18] is mainly used for data with dense feature learning such as images and speech, while RNN and Long Short-Term Memory (LSTM) are popular choices in text homogenization and serialization of high-dimensional sparse features [19]. Driving behavior recognition involves classifying time series data captured from inertial sensors such as 3-axis accelerometers or gyroscopes. Recently, CNN has established itself as a powerful technique for activity recognition, where convolution and pooling operations were applied along the temporal dimension of sensor signals [20]. Furthermore, in most of the state-of-the-art works on CNN for activity recognition, 1D/2D convolution was employed in individual time series to capture local dependency along the temporal dimension of sensor signals [21,22]. The combination of CNN and LSTM had already offered state-of-the-art results in speech recognition, wearable activity recognition, online defect recognition of CO_2_ welding, etc., where modeling temporal information was required [14,23,24,25,26]. This kind of architecture was able to capture time dependencies on features extracted by convolution operations. In this work, we focused on extracting key features using an end-to-end deep learning approach without the requirement of feature selection. In addition, features characterizing both driving behaviors and automotive running were used to represent a driver’s personality.

### 2.2. Problem Formulation

#### 2.2.1. In-Vehicle CAN-BUS Sensor Data Preparation and Analysis

Our models are evaluated on Ocslab driving dataset [27,28]. The dataset is used for the AI/ML based driver classification challenge track in the 2018 Information Security R&D dataset challenge held in South Korea [29]. The dataset holds a total of 94,401 records, which are created from an experiment where ten drivers labeled from “A” to “J” completed two round trips in a similar time zone from 8 p.m. to 11 p.m. on weekdays. The On Board Diagnostics 2 (OBD-II) and CarbigsPare are used as OBD-II scanner for data collection at 1 Hz sampling rate.

**Figure 2:**
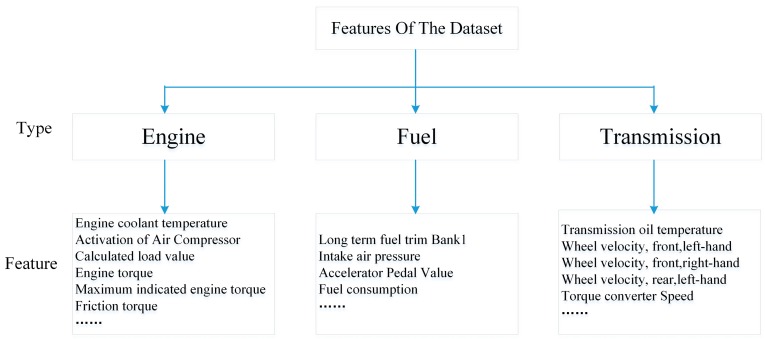
The data structure of Ocslab driving dataset.

Originally, there are 51-Dimensional (51D) features in the dataset and the data structure of Ocslab driving dataset is depicted in Figure 2.

**Figure 3:**
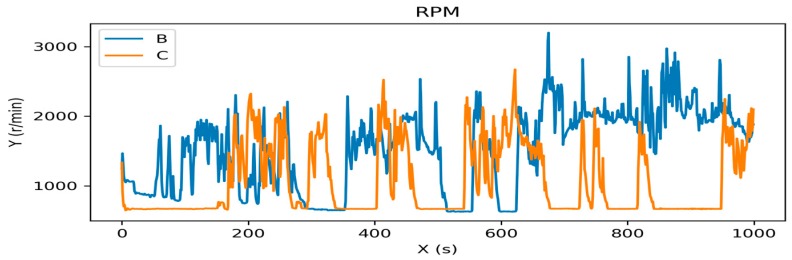
The visualization of revolutions per minute (RPM) of drivers B and C.

Some features are visualized in driver’s driving pattern. Figure 3 shows the difference of revolutions per minute (RPM) when drivers B and C drove the car in the experiment.

#### 2.2.2. Data Processing

This work used all 51 original features in the dataset without complex feature selection. Before feeding to our classification model, the data was normalized and processed using sliding window technique. The input data is defined as $\chi \in {\mathbb{R}}^{N_{\chi }\times M_{\chi }}$. The data at time step t is defined as:

(1) $$\chi_{t}={\left(\chi_{t}^{1},\chi_{t}^{2},\ldots ,\chi_{t}^{N_{x}-1},\chi_{t}^{N_{x}}\right)}^{T}$$

where $N_{\chi }$ denotes the dimensionality of $\chi_{t}$, and $M_{\chi }$ represents the amount of dataset χ, that is, the total number of χ in all time steps.

Since the scales of features in the dataset are different, they are needed to be normalized in a classification algorithm. Specifically, the normalization process for unifying data scales is defined as:

(2) $$\begin{array}{l} {\bar{\chi }}_{t}^{n}=\frac{\left(\chi_{t}^{n}-mean\left(\chi^{n}\right)\right)}{std\left(\chi^{n}\right)}\\ {\bar{\chi }}_{t}={\left({\bar{\chi }}_{t}^{1},{\bar{\chi }}_{t}^{2},\ldots ,{\bar{\chi }}_{t}^{N_{x}-1},{\bar{\chi }}_{t}^{N_{x}}\right)}^{T} \end{array}$$

where $mean\left(\chi^{n}\right)$ and $std\left(\chi^{n}\right)$ represent the mean and standard deviation of the $n^{th}$ dimension of dataset χ, respectively.

Driving behavior is a continuous process, so sliding window technique is adopted to divide the entire data set into multiple discrete data segments by time period. In order to extract contextual features and ensure the continuity of data segments, presuming $T_{x}$ is window size, data segments are extracted by the sliding window method with overlapping window. For the dataset with $N_{\chi }$ dimensions, the windowed sample $x_{i}$ holds $D_{x}=T_{x}\times N_{x}$ dimensions, which are generated by

(3) $$x_{i}={\left({\bar{\chi }}_{t-T_{\chi }+1},{\bar{\chi }}_{t-T_{\chi }+2},\ldots ,{\bar{\chi }}_{t}\right)}^{T}\in {\mathbb{R}}^{N_{x}},\left(t=T_{\chi },T_{\chi }+\Delta t,T_{\chi }+2\Delta t,\ldots \right)$$

**Figure 4:**
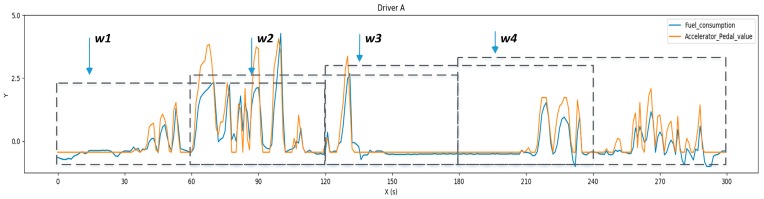
Overlapping sliding window method. Four windows ($w_{1}$, $w_{2}$, $w_{3}$, $w_{4}$) were obtained from the 300 samples when setting the window size $T_{x}$ to 120 samples and time step Δt to 60 samples.

As shown in Figure 4, the windowing dataset $X\in {\mathbb{R}}^{N_{x}\times M_{x}}$ is generated when $x_{i}$ moves at the time axis by the time step Δt, where $N_{x}=N_{\chi }$ and $M_{x}$ is the amount of the windowing dataset X.

### 2.3. Our Proposed Framework

#### 2.3.1. Main Procedure of Our Proposed Architecture

**Figure 5:**
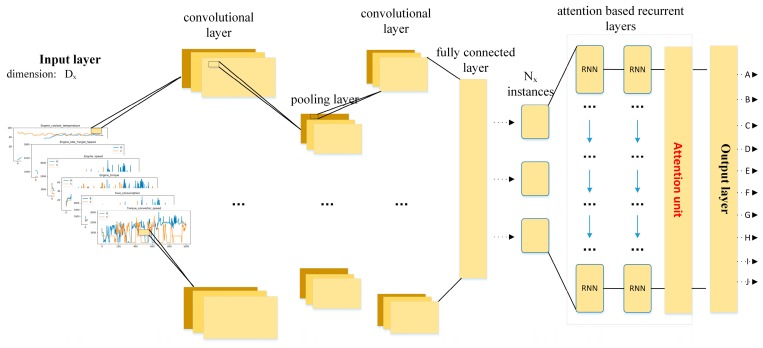
The main procedure of our proposed model.

Compared to the structure of DeepConvLSTM proposed in [14,23,24,25,26], we introduce an attention mechanism in [30], and redesign the convolutional and recurrent layer referring to [31,32]. As shown in Figure 5, the proposed model for driving behavior identification using in-vehicle CAN-BUS sensor data consists of an input layer, middle layers and a classifier layer. $D_{x}$ is the dimension of input data sample in input layer and $N_{y}$ is the output categories in output layer.

The middle layers consist of convolutional layers, pooling layers, recurrent layers and a fully connected layer.

Figure 5 shows the flowchart of our model. First, a window series extracted from the CAN-BUS sensor data is passed into convolutional layers. Next, attention-based recurrent layers are used for time series feature extraction, whose inputs are the feature maps of the last convolutional layer. Lastly, the output layer, followed by the recurrent layers, is used to yield class probability distribution for driving behavior identification.

#### 2.3.2. Convolutional and Pooling Layers for Feature Extraction

Our model contains depth-wise separable convolutional layers [33] and a pooling layer in the beginning, which take convolutional operations on the input time series data. Each group of outputs of a convolutional layer is called feature map, which are regarded as features extracted from input signals. It is supposed that the number of feature map from the ${\left(l-1\right)}^{th}$ convolutional layer is $n_{l-1}$, and the size of each feature map is $m_{l-1}=w_{l-1}\times h_{l-1}$. The total number of neurons in the $l-1^{th}$ layer is $n_{l-1}\times m_{l-1}$. The $k^{th}$ feature map output from the $l^{th}$ convolutional layer is:

(4) $$X^{\left(l,k\right)}=\sigma \left(\sum_{p=1}^{n_{l-1}}W^{\left(l,k,p\right)}⊗X^{\left(l-1,p\right)}+b^{\left(l,k\right)}\right)$$

where σ is the ReLU activation function, $W^{\left(l,k,p\right)}\in {\mathbb{R}}^{u\times v}$, which is the 2D filter mapping from the $p^{th}$ feature map of the $l-1^{th}$ layer to the $k^{th}$ feature map of the $l^{th}$ layer. In addition, $X^{\left(l,k\right)}\in {\mathbb{R}}^{w_{l}\times h_{l}}$, $w_{l}=w_{l-1}-w_{f}+1$, $h_{l}=h_{l-1}-h_{f}+1$, where $w_{f}$ and $h_{f}$ are the width and height of the filter, respectively.

Generally, the convolutional layers are followed by pooling operations, which could greatly reduce the dimension of feature maps and avoid over-fitting. The output of the pooling layer is as follow:

(5) $$X^{\left(l+1\right)}=down\left(X^{\left(l\right)}\right)$$

where $down(X^{l})$ is down-sampling function for the $l^{th}$ convolutional layer $X^{\left(l\right)}$, which generally takes the maximum (Maximum Pooling) or average (Average Pooling) of all neurons in pooling region.

From equation (4) and equation (5), we can see that the first convolutional layer operates sensor data with $D_{x}$ dimensions into $c_{f_{1}}\times m_{f_{1}}$ feature maps by applying 2D filters with shape $\left[h_{f_{1}},w_{f_{1}},c_{f_{1}},m_{f_{1}}\right]$, where $h_{f_{1}}$, $w_{f_{1}}$, $c_{f_{1}}$, $m_{f_{1}}$ are respectively the filter height, filter width, input channel and channel multiplier of the $1^{st}$ convolutional layer. The following pooling layer uses a kernel with shape $\left[1,h_{k_{1}},w_{k_{1}},1\right]$ to down-sample feature maps, where $h_{k_{1}}$, $w_{k_{1}}$ are respectively the $1^{st}$ pooling layer kernel height and width.

The window inputs are split into $N_{x}$ instances in time dimension. This $N_{x}$ instances data is then fed into recurrent layers, in which each layer owns $N_{h}$ hidden nodes.

#### 2.3.3. Attention Based Recurrent Layer

There are two extended Recurrent Neural Network (RNN): Long Short-Term Memory (LSTM) [34] and Gated Recurrent Unit (GRU) [35]. They all use purpose-built memory cells to store information, which is helpful to find and exploit long range dependencies in time series data and thus can be further leading to more efficient driving pattern recognition. Thus, LSTM and GRU are adopted as the recurrent components that make use of the concept of gating, a mechanism based on the component-wise multiplication of inputs, which defines the behavior of each individual memory cell and decides whether to retain the state of the last moment or not, as well as to receive external inputs at this moment. LSTM is done with forget gates and input gates while GRU adopts update gates.

**Figure 6:**
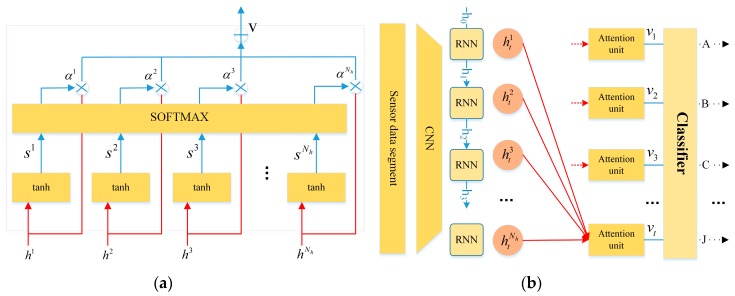
The attention unit.

Time series sensor data contains more complex temporal information. Not all feature maps have the equal contribution in the identification of driving behaviors. With an attention mechanism, encoding the full input sequences into a fixed-length vector is no longer required. Thus the attention mechanism (see Figure 6) introduced by [30] is extended to capture salient structures of data, extracting more valuable feature maps than others for classification. The attention unit can also be viewed as a weighted average of output over time, where the weights could be learned automatically through context.

As depicted in Figure 6a, the attention unit takes input vector $\left\{h^{1},\ldots ,h^{N_{h}}\right\}$, which is the hidden state of the recurrent layer, and outputs a contextual attention-based vector v, which is a weighted arithmetic mean of the input vector where the weights are learned based on the importance of each element of the vector. As depicted in Figure 6b, the output of the attention model $v_{t}$, which remains the importance of the representation of feature maps, is used as the input vector for the following classifier.

For each segment feature $x^{i}$ at $t^{th}$ time step, the context information is calculated by:

(6) $$\left\{ \begin{array}{l} s_{t}^{i}=W_{s}\tanh\left(W_{h}h_{t}^{i}+b_{s}\right)\\ \alpha_{t}^{i}=\frac{\exp\left(s_{t}^{i}\right)}{\sum_{i=1}^{N_{x}}\exp\left(s_{t}^{i}\right)},\sum_{i=1}^{N_{x}}\alpha_{t}^{i}=1 \end{array}\right.$$

where $W_{s}$, $W_{h}$ and $b_{s}$ are parameters to be learned, $\alpha_{t}^{i}$ is the attention weight at $t^{th}$ time step describing the importance of the input vector. Given the current hidden state $h_{t}$ of the decoder, it returns un-normalized score $s_{t}^{i}$. Once the scores *S_t_* for all the nodes $\left\{h^{1},\ldots ,h^{N_{h}}\right\}$ are computed, the RNN is able to obtain $\alpha_{t}^{i}$ at $t^{th}$ time step. The contextual attention-based output is:

(7) $$v_{t}=\sum_{i=1}^{N_{h}}\alpha_{t}^{i}h_{t}^{i}$$

where $v_{t}$ represents context vector which is a dynamic representation of the feature map at $t^{th}$ time step.

Next, $v_{t}$ is augmented to the basic LSTM and the basis formulation of LSTM [34] is below:

(8) $$\left\{ \begin{array}{l} i_{t}=\sigma \left(W_{xi}x_{t}+W_{hi}h_{t-1}+W_{ci}c_{t-1}+b_{i}\right)\\ f_{t}=\sigma \left(W_{xf}x_{t}+W_{hf}h_{t-1}+W_{cf}c_{t-1}+b_{f}\right)\\ c_{t}=f_{t}c_{t-1}+i_{t}\tanh\left(W_{xc}x_{t}+W_{hc}h_{t-1}+b_{c}\right)\\ o_{t}=\sigma \left(W_{xo}x_{t}+W_{ho}h_{t-1}+W_{co}c_{t}+b_{o}\right)\\ h_{t}=o_{t}\tanh\left(c_{t}\right)\\ v_{t}=\sum \alpha_{t}h_{t} \end{array}\right.$$

where σ is logistic sigmoid function, and i, f, o and c are respectively the input gate, forget gate, output gate, and cell input activation vectors, which are the same size as the hidden vector h and could be updated at every time step *t*. $W_{hi}$ is the weight matrix of hidden-input gate and $W_{xo}$ is the matrix of input–output gate.

Similarly, $v_{t}$ is added into GRU referred to [35] and the outputs are calculated by:

(9) $$\left\{ \begin{array}{l} z_{t}=\sigma \left(W_{z}x_{t}+U_{z}h_{t-1}+b_{z}\right)\\ r_{t}=\sigma \left(W_{t}x_{t}+U_{t}h_{t-1}+b_{r}\right)\\ {\bar{h}}_{t}=\tanh\left(Wx_{t}+U\left(r_{t}\circ h_{t-1}\right)\right)\\ h_{t}=\left(1-z_{t}\right)\circ h_{t-1}+z_{t}\circ {\bar{h}}_{t}\\ v_{t}=\sum \alpha_{t}h_{t} \end{array}\right.$$

where ∘ is an element-wise multiplication, $z_{t}$, $r_{t}$, ${\bar{h}}_{t}$ and $h_{t}$ are the update gate, reset gate, candidate activation and output activation, respectively.

#### 2.3.4. Classifier Layer for Driving Behavior Identification

Then the output of recurrent layer $X_{r}=\left\{x^{1},\ldots ,x^{N_{h}}\right\}$ is fed into a classifier layer to generate the prediction $\hat{y}$. In the classifier layer, a learnable matrix $W_{o}$ with a bias term $b_{o}$ are used to decode $X_{r}$ into $\hat{y}$, such that $\hat{y}=W_{o}X_{r}+b_{o}$. Therefore, the classifier layer is a fully connected layer with sharing parameter $W_{o}$ and $b_{o}$.

### 2.4. Model Training

(A) Learning:

Since our model is a multi-class classification model, the most commonly used objective function is cross-entropy cost function, which is similar to the K-L divergence between two distributions:

(10) $$J\left(w\right)=-\sum_{i=1}^{m}\left(y^{\left(i\right)}\log\left(\hat{y}\left(x^{\left(i\right)}\right)\right)+\left(1-y^{\left(i\right)}\right)\log\left(1-\hat{y}\left(x^{\left(i\right)}\right)\right)\right)$$

where $\left(x^{\left(i\right)},y^{\left(i\right)}\right)$ represents the input sample with label i, and $\hat{y}\left(x^{\left(i\right)}\right)$ is the prediction of the instance $x^{\left(i\right)}$.

(B) Overfitting:

An overfitting model performs poorly since it overreacts to the given training data. Therefore, dropout is adopted to DeepConvLSTM/DeepConvGRU framework.

### 2.5. Model Evaluation

In order to compare our models with the state-of-the-art methods, three evaluation metrics are selected to evaluate our experiments: Accuracy, AUC [36] and weighted F1 score. Previous related work used the weighted F1 score as the primary performance metric [14]. The weighted F1 score is defined as:

(11) $$F1=\sum_{i}2*\omega_{i}\frac{precision_{i}\cdot recall_{i}}{precision_{i}+recall_{i}}$$

where i is class index and $\omega_{i}=n_{i}/N$ is the proportion of samples of the class i, with $n_{i}$ being the number of samples of the $i^{th}$ class and N being the total number of samples.

## 3. Results

Our model is evaluated and compared with other two methods, which are variants of our model created by removing the attention units from our model. It is also compared with some other state-of-the-art models [27], which are described below:

**DeepConvGRU–Attention:** This model has two depth wise separable convolutional layers and a pooling layer in CNN module, followed by stacked GRU with two attention-based layers.

**DeepConvLSTM–Attention:** Compared to DeepConvGRU–Attention, this model replaces GRU with LSTM in the recurrent layers.

**DeepConvGRU:** This model is similar to DeepConvGRU–Attention without attention units in model training.

**DeepConvLSTM:** Similarly, this model removes attention units from DeepConvLSTM–Attention.

**CNN:** This model owns two depth wise separable convolutional layers and a pooling layer with a softmax classifier in the output. The baseline algorithm is used to verify the effectiveness of the recurrent layers in finding and exploiting long range dependencies in time series data, which is suitable for driving pattern recognition.

**LSTM:** This model has two stacked LSTM layers, referred to in [37,38].

**DNN:** This model has two stacked hidden layers, referred to in [38].

Our model used all original 51D features to identify driving behaviors. To show the power of our end-to-end framework, feature selection referred to in [27] was implemented, selecting 15-Dimensional (15D) features from the original 51D features and deriving three statistical features for original features. In total, statistical 45-Dimensional (45D) features were obtained. Table 1 shows the selected original features and statistical features. We chose the KNN, Decision Tree and Random Forest algorithms as the baselines [27,38] as they have been proven to yield good performance.

The Ocslab driving dataset was split into a training set and a test set with a ratio of 7:3 for validating the model performance. To achieve the best performance for each model in the dataset, the parameters of models were fully tuned. The hyper-parameters of compared deep models are listed in Table 2, which shows the structure of layers, window size, dropout, activation function and optimizer.

The window size $T_{x}$ and Δt were set referred to [39]. As shown in Table 3, for different models, different features were chosen to get the best performance.

**Figure 7:**
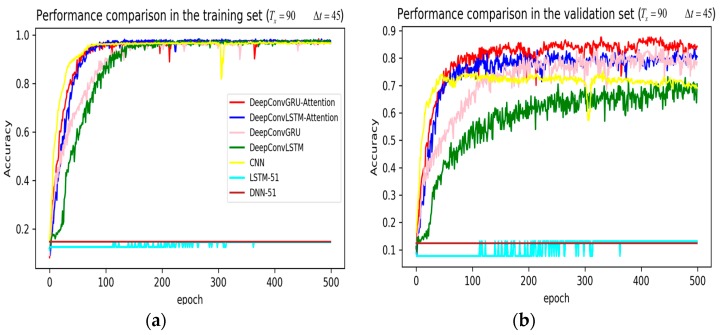
Performance comparison of different models in accuracy using 51 original features when setting $T_{x}$ to 90 samples and Δt to 45 samples. (**a**) Performance comparison in the training stage. (**b**) Performance comparison in the validation stage.

**Figure 8:**
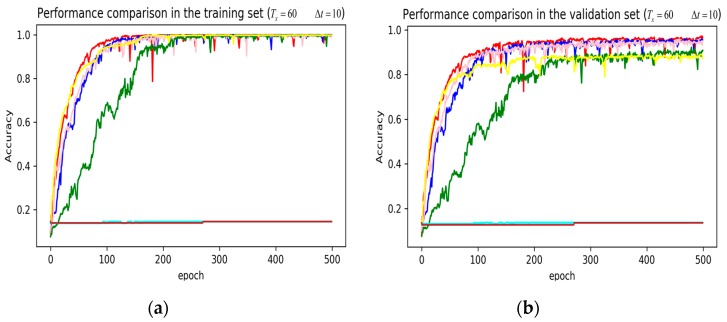
Performance comparison of different models in accuracy using 51 original features when setting $T_{x}$ to 60 samples and Δt to 10 samples. (**a**) Performance comparison in the training stage. (**b**) Performance comparison in the validation stage.

**Figure 9:**
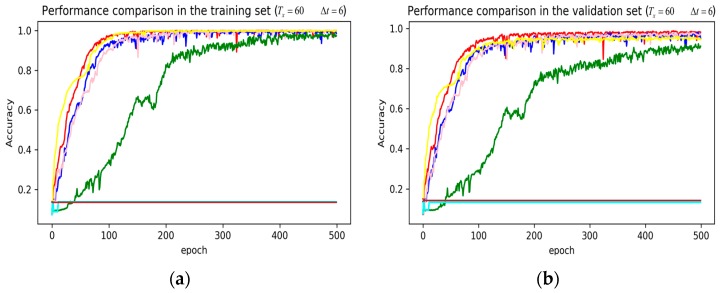
Performance comparison of different models in accuracy using 51 original features when setting $T_{x}$ to 60 samples and Δt to 6 samples. (**a**) Performance comparison in the training stage. (**b**) Performance comparison in the validation stage.

**Figure 10:**
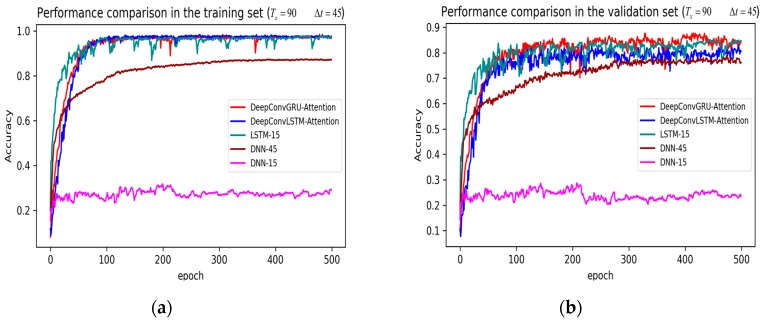
Performance comparison of different models in accuracy using 51 original features when setting $T_{x}$ to 90 samples and Δt to 45 samples. (**a**) Performance comparison in the training stage. (**b**) Performance comparison in the validation stage. Here, DeepConvGRU and DeepConvLSTM using the original 51D features performed similar to LSTM and DNN using the selected 15D features and statistical 45D features. In addition, DNN using selected 15D features also could not be converged.

**Figure 11:**
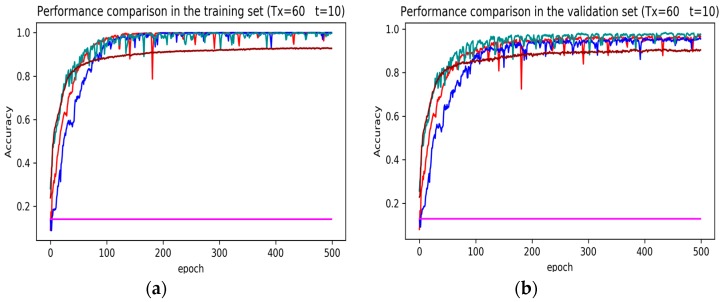
Performance comparison of different models in accuracy using original 51D features when setting $T_{x}$ to 60 samples and Δt to 10 samples. (**a**) Performance comparison in the training stage. (**b**) Performance comparison in the validation stage. Here, the attention-based DeepConvGRU and DeepConvLSTM performed better than DNN using statistical 45D features.

**Figure 12:**
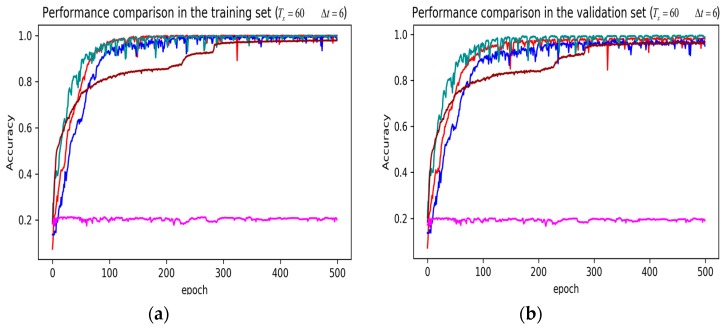
Performance comparison of different models in accuracy using original 51D features when setting $T_{x}$ to 60 samples and Δt to 6 samples. (**a**) Performance comparison in the training stage. (**b**) Performance comparison in the validation stage. Here, the attention-based DeepConvGRU and DeepConvLSTM also yielded good performance.

In training stage, the effects of the attention and RNN units were investigated in terms of model learning efficiency and generalization ability under Adam optimizer. The 5-fold cross-validation was used to make sure the proposed model was generalized over the dataset, in which the total data samples were divided into five parts, where four of them were used for the training model and the remaining one was employed for validation. Figure 7, Figure 8, Figure 9, Figure 10, Figure 11 and Figure 12 illustrate the evaluations of the first fold training and the verification stage with respect to accuracy.

The legends of Figure 7, Figure 8 and Figure 9 are identical. From Figure 7a, LSTM and DNN could not be converged if using all the original features without performing any feature selection. Other models could automatically select features because of the convolutional layers. From Figure 7b, DeepConvGRU and DeepConvLSTM gained better generalization ability, capturing local dependency among the temporal dimension compared with CNN. DeepConvGRU yielded faster learning efficiency than DeepConvLSTM because GRU has less parameters and therefore was easier to be converged. In Figure 8 and Figure 9, it can be seen that the attention based DeepConvGRU and DeepConvLSTM also performed the best compared with other models. The attention mechanism made the model easier to be converged.

From the results in Figure 7, Figure 8 and Figure 9, we can see that the attention based DeepConvGRU/DeepConvLSTM consistently outperformed the baselines. It can be noticed that DeepConvGRU made a striking performance improvement. This may be because that LSTM has more parameters than GRU, which makes it more difficult to be converged on a small dataset. The fact that DeepConvGRU/DeepConvLSTM obtained better performance than CNN may be due to the ability of RNN cells to capture temporal dynamics within the data sequences. However, the baseline CNN was only capable of modelling time sequences up to the length of the kernels. Moreover, LSTM and DNN could not be converged if using all original features. So LSTM and DNN with selected 15D features and statistical 45D features were investigated and compared with other models in Figure 10, Figure 11 and Figure 12.

The legends of Figure 10, Figure 11 and Figure 12 are identical. From Figure 10, Figure 11 and Figure 12, it can be seen that the attention based DeepConvGRU and DeepConvLSTM using original 51D features without any feature selection gained similar good performance to LSTM and DNN using artificially designed features. The baseline DNN using statistical 45D features yielded poor learning efficiency and generalization ability when setting $T_{x}$ to 60 samples and Δt to 10 samples. Furthermore, the baseline DNN using selected 15D features could not be converged in all cases.

To fully show the performance comparison of the models, F1 scores of the models were explored except for the models that could not be converged. The results are shown in Table 4.

Table 4 and Table 5 illustrates the performance comparison of the proposed four variants of our framework compared with traditional models including CNN, LSTM, KNN, Decision Tree and Random Forest under different $T_{x}$ and Δt. Experimental results showed that our framework outperformed traditional methods without any feature selection. Without feature selection, our framework also performed better than DNN and gained similar good performance to LSTM using artificially designed features. Moreover, the attention-based DeepConvGRU and DeepConvLSTM–Attention yielded better improvements than DeepConvGRU and DeepConvLSTM, respectively. In conclusion, the attention mechanism effectively helps to learn more discriminative features in time series data.

## 4. Discussion

From the performance comparison of our attention based DeepConvGRU/DeepConvLSTM with the baseline models without RNN unit and attention unit in the dense layer, several main findings were obtained.

First, DeepConvGRU/DeepConvLSTM reaches a higher F1 score. It is significantly more suitable for identifying disambiguate closely-related activities, which tend to differ with ordering time series data, and it is applicable for the activities that are longer than the observation window. The experimental results show that our framework can capture local dependency among the temporal dimension as well as across spatial locations.

Second, the attention mechanism makes DeepConvGRU/DeepConvLSTM gaining better generalization ability, which could automatically learn the weights of features and extract important features for the driving behavior identification.

Third, our framework outperforms traditional methods without any feature selection. Since CAN-BUS data sometimes are massive and high-dimensional, our framework is very advantageous in the case of difficult feature selection.

Furthermore, since the driving activity duration is longer than the sliding window size, experimental results showed that the model can nevertheless obtain a good performance. This might be because long driving activities are made of several short characteristic patterns, allowing the model to spot and classify the driving activity even without a complete view of the activity.

## 5. Conclusions

This paper presented a deep learning framework based on the combination of CNN and GRU/LSTM recurrent network to identify driving behaviors using in-vehicle CAN-BUS sensor data. In the framework, the GRU/LSTM cells were integrated into CNN to distinguish activities from similar driving behaviors. The attention based DeepConvGRU/DeepConvLSTM took advantage of learning temporal dynamics. Experimental results showed that our proposed method outperformed the traditional methods on the Ocslab driving dataset.

From the experimental results, it was also obvious that the proposed framework is able to learn features from original signals and fuse the learned features without any specific preprocessing. Surprisingly but reasonably, the attention-based DeepConvGRU achieved competitive F1 scores (0.984 and 0.970 respectively) while directly using 51-channel original sensor data. This provided a path to address a similar issue that sensor data from different sources must be automatically processed.

In the future, further researches can be conducted in the following aspects:

First, a multi-scale approach should be developed to achieve accurate activity recognition on in-vehicle CAN-BUS sensor data.

Second, due to the individual privacy protection of some driving datasets, most datasets do not disclose the complete time series data of driving behaviors from different drivers. Therefore, our framework can only be verified on a public driving behavior dataset. In the future, we need to investigate our model on more practical large-scale Naturalistic Driving Studies (NDS) datasets, such as 100-CAR [40], SHRP2 NDS [41,42], etc.

## Figures and Tables

**Table 1:** Selected original features and statistical features.

Selected 15D Features	Statistical 45D Features
‘Long term fuel trim bank’, ’Intake air pressure’, ‘Accelerator pedal value’, ‘Fuel consumption’, ‘Friction torque’, ‘Maximum indicated engine torque’, ‘Engine torque’, ‘Calculated load value’, ‘Activation of air compressor’, ‘Engine coolant temperature’, ‘Transmission oil temperature’, ‘Wheel velocity front left-hand’, ‘Wheel velocity front right-hand’, ‘Wheel velocity rear left-hand’, ‘Torque converter speed’	Mean Median Standard deviation

**Table 2:** Hyper-parameters of models on Ocslab driving dataset.

Model	Layers (l) ^1^	Dropout	Activation Function	Optimizer
DeepConvGRU–Attention	(1 × 60) − 1 × 20 − 1 × 6 − 128 − 128 − 10	0.5	ReLU	Adam
DeepConvLSTM–Attention	(1 × 60) − 1 × 20 − 1 × 6 − 128 − 128 − 10	0.5	ReLU	Adam
DeepConvGRU	(1 × 60) − 1 × 20 − 1 × 6 − 128 − 128	0.5	ReLU	Adam
DeepConvLSTM	(1 × 60) − 1 × 20 − 1 × 6 − 128 − 128	0.5	ReLU	Adam
CNN	(1 × 60) − 1 × 20 − 1 × 6	0.5	ReLU	Adam
LSTM	128 − 128	0.5	ReLU	Adam
DNN	1000 − 1000	0.5	ReLU	Adam

^1^ ‘(1*60)’ represents the kernel size of input-to-state convolutional layer. ‘1*20’ and ‘1*6’ represent the corresponding kernel sizes of state-to-state convolutional layer and pooling layer. ‘128’ refers to the number of hidden states in the recurrent layers while ‘10’ represents the size of attention vector. ‘1000’ refers to the number of hidden states in the hidden layers of DNN.

**Table 3:** Hyper-parameters of models on Ocslab driving dataset.

Model	$\mathrm{Window}\;\mathrm{Size}\;(T_{x})$	Time Step (Δt)	Selected Features
DeepConvGRU-Attention	90/60/60	45/10/6	original 51D features
DeepConvLSTM-Attention	90/60/60	45/10/6	original 51D features
DeepConvGRU	90/60/60	45/10/6	original 51D features
DeepConvLSTM	90/60/60	45/10/6	original 51D features
CNN	90/60/60	45/10/6	original 51D features
LSTM-51	90/60/60	45/10/6	original 51D features
LSTM-15	90/60/60	45/10/6	selected 15D features
DNN-51	90/60/60	45/10/6	original 51D features
DNN-15	90/60/60	45/10/6	selected 15D features
DNN-45	90/60/60	45/10/6	statistical 45D features
KNN	90/60/60	45/10/6	statistical 45D features
Decision Tree	90/60/60	45/10/6	statistical 45D features
Random Forest	90/60/60	45/10/6	statistical 45D features

**Table 4:** Driving behavior identification for different methods when setting $T_{x}$ to 60 samples and Δt to 10 samples.

Model	$T_{x}=60,\;\Delta t=10$					
	Accuracy		AUC		F1 Score	
	Mean	Std	Mean	Std	Mean	Std
KNN	0.812	0	0.8986	0	0.8157	0
DecisionTree	0.7432	0.0966	0.8638	0.0502	0.7402	0.1002
RandomForest	0.7049	0.0589	0.8541	0.0275	0.7565	0.0485
DeepConvGRU-Attention	0.9701	0.0052	0.9959	0.0007	0.9702	0.0052
DeepConvLSTM-Attention	0.9524	0.0138	0.9946	0.0015	0.9526	0.0137
DeepConvGRU	0.9565	0.0091	0.9959	0.0011	0.9563	0.0093
DeepConvLSTM	0.905	0.0062	0.9887	0.0008	0.9047	0.0064
CNN	0.9081	0.0096	0.993	0.0013	0.908	0.0096
LSTM-15	0.9774	0.012	0.9982	0.0008	0.9771	0.0124
DNN-45	0.9188	0.0301	0.9731	0.0164	0.9145	0.0337

**Table 5:** Driving behavior identification for different methods when setting $T_{x}$ to 60 samples and Δt to 6 samples.

Model	$T_{x}=60,\;\Delta t=6$					
	Accuracy		AUC		F1 Score	
	Mean	Std	Mean	Std	Mean	Std
KNN	0.9033	0	0.947	0	0.9045	0
DecisionTree	0.8543	0.0062	0.9213	0.004	0.8537	0.0069
RandomForest	0.8739	0.0044	0.9359	0.0025	0.8934	0.0034
DeepConvGRU-Attention	0.9836	0.0015	0.9978	0.001	0.9836	0.0015
DeepConvLSTM-Attention	0.9786	0.0068	0.9978	0.0006	0.9787	0.0068
DeepConvGRU	0.9772	0.0062	0.9968	0.0008	0.9772	0.0062
DeepConvLSTM	0.9519	0.0186	0.9944	0.0013	0.9497	0.019
CNN	0.9568	0.0072	0.9984	0.0002	0.9567	0.0073
LSTM-15	0.993	0.0015	0.9996	0.0001	0.9929	0.0015
DNN-45	0.9395	0.0358	0.9682	0.0281	0.9315	0.0493

## References

[1] Enev M., Takakuwa A., Koscher K., Kohno T. (2016). Automobile Driver Fingerprinting. Proc. Priv. Enhanc. Technol..

[2] Nishiwaki Y., Ozawa K., Wakita T., Miyajima C., Itou K., Takeda K. (2007). Driver identification based on spectral analysis of driving behavioral signals. Advances for in-Vehicle and Mobile Systems.

[3] Wahab A., Quek C., Tan C.K., Takeda K. (2009). Driving profile modeling and recognition based on soft computing approach. IEEE Trans. Neural Netw..

[4] Zhang X., Zhao X., Rong J. (2014). A study of individual characteristics of driving behavior based on hidden markov model. Sens. Transducers.

[5] Kaplan S., Guvensan M.A., Yavuz A.G., Karalurt Y. (2015). Driver Behavior Analysis for Safe Driving: A Survey. IEEE Trans. Intell. Transp. Syst..

[6] Choi S., Kim J., Kwak D., Angkititrakul P., Hansen J.H. Analysis and classification of driver behavior using in-vehicle can-bus information. Proceedings of the ITS World Congress 2012.

[7] Kedar-Dongarkar G., Das M. (2012). Driver classification for optimization of energy usage in a vehicle. Procedia Comput. Sci..

[8] Van Ly M., Martin S., Trivedi M.M. Driver classification and driving style recognition using inertial sensors. Proceedings of the Intelligent Vehicles Symposium IEEE.

[9] Liu H., Taniguchi T., Tanaka Y., Takenaka K., Bando T. (2017). Visualization of Driving Behavior Based on Hidden Feature Extraction by Using Deep Learning. IEEE Trans. Intell. Transp. Syst..

[10] Liu H.L., Taniguchi T., Takano T., Tanaka Y. Visualization of driving behavior using deep sparse autoencoder. Proceedings of the Intelligent Vehicles Symposium IEEE.

[11] Fazeen M., Gozick B., Dantu R., Bhukhiya M., González M.C. (2012). Safe Driving Using Mobile Phones. IEEE Trans. Intell. Transp. Syst..

[12] Dai J., Teng J., Bai X., Shen Z., Xuan D. Mobile phone based drunk driving detection. Proceedings of the 4th International Conference on Pervasive Computing Technologies for Healthcare.

[13] Zaldivar J., Calafate C.T., Cano J.C., Manzoni P. Providing accident detection in vehicular networks through OBD-II devices and Android-based smartphones. Proceedings of the IEEE Conference on Local Computer Networks.

[14] Ordóñez F.J., Roggen D. (2016). Deep convolutional and lstm recurrent neural networks for multimodal wearable activity recognition. Sensors.

[15] Miyajima C., Nishiwaki Y., Ozawa K., Wakita T., Itou K., Takeda K., Itakura F. (2007). Driver modeling based on driving behavior and its evaluation in driver identification. Proc. IEEE.

[16] Wakita T., Ozawa K., Miyajima C., Igarashi K., Itou K., Takeda K., Itakura F. Driver identification using driving behavior signals. Proceedings of the IEEE Intelligent Transportation Systems Conference.

[17] Meng X., Lee K.K., Xu Y. Human Driving Behavior Recognition Based on Hidden Markov Models. Proceedings of the IEEE International Conference on Robotics & Biomimetics.

[18] Krizhevsky A., Sutskever I., Hinton G.E. ImageNet classification with deep convolutional neural networks. Proceedings of the International Conference on Neural Information Processing Systems.

[19] Foland W., Martin J.H. CU-NLP at SemEval-2016 Task 8: AMR Parsing using LSTM-based Recurrent Neural Networks. Proceedings of the International Workshop on Semantic Evaluation.

[20] Ha S., Choi S. Convolutional neural networks for human activity recognition using multiple accelerometer and gyroscope sensors. Proceedings of the International Joint Conference on Neural Networks.

[21] Duffner S., Berlemont S., Lefebvre G., Garcia C. 3D gesture classification with convolutional neural networks. Proceedings of the IEEE International Conference on Acoustics, Speech and Signal Processing.

[22] Zeng M., Nguyen L.T., Yu B., Mengshoel O.J., Zhu J., Wu P., Zhang J. Convolutional Neural Networks for Human Activity Recognition using Mobile Sensors. Proceedings of the International Conference on Mobile Computing, Applications and Services.

[23] Sainath T.N., Vinyals O., Senior A., Sak H. Convolutional, long short-term memory, fully connected deep neural networks. Proceedings of the IEEE International Conference on Acoustics.

[24] Bilgera C., Yamamoto A., Sawano M., Matsukura H., Ishida H. (2018). Application of Convolutional Long Short-Term Memory Neural Networks to Signals Collected from a Sensor Network for Autonomous Gas Source Localization in Outdoor Environments. Sensors.

[25] Liu T., Bao J., Wang J., Zhang Y. (2018). A Hybrid CNN–LSTM Algorithm for Online Defect Recognition of CO_2_ Welding. Sensors.

[26] Nguyen V., Nguyen M., Choi J., Kim Y. (2018). NLOS Identification in WLANs Using Deep LSTM with CNN Features. Sensors.

[27] Kwak B.I., Woo J., Kim H.K., Huy K. Know your master: Driver profiling-based anti-theft method. Proceedings of the 14th Annual Conference on Privacy, Security and Trust.

[28] Driving Dataset. http://ocslab.hksecurity.net/Datasets/driving-dataset.

[29] AI/ML Based Driver Classification Challenge Track. http://datachallenge.kr/challenge18/vehicle/introduction/.

[30] Attention Mechanism. https://blog.heuritech.com/2016/01/20/attention-mechanism/.

[31] Li X. cnnPlusLSTM. https://github.com/lixiaoyu0575/cnnPlusLSTM.

[32] Saeed A. Implementing a CNN for Human Activity Recognition in Tensorflow. http://aqibsaeed.github.io/2016-11-04-human-activity-recognition-cnn/.

[33] Chollet F. Xception: Deep Learning with Depthwise Separable Convolutions. Proceedings of the 2017 IEEE Conference on Computer Vision and Pattern Recognition.

[34] Graves A. (2013). Generating sequences with recurrent neural networks. arXiv.

[35] Chung J., Gulcehre C., Cho K.H., Bengio Y. (2014). Empirical evaluation of gated recurrent neural networks on sequence modeling. arXiv.

[36] Fawcett T. (2005). An introduction to ROC analysis. Pattern Recogn. Lett..

[37] Jiménez D., Hernández S., Fraile-Ardanuy J., Serrano J., Fernández R., Álvarez F. (2018). Modelling the Effect of Driving Events on Electrical Vehicle Energy Consumption Using Inertial Sensors in Smartphones. Energies.

[38] Wang Y., Ho I.W. Joint Deep Neural Network Modelling and Statistical Analysis on Characterizing Driving Behaviors. Proceedings of the 2018 IEEE Intelligent Vehicles Symposium (IV).

[39] Carvalho E., Ferreira B.V., Ferreira J., De Souza C., Carvalho H.V., Suhara Y., Pentland A.S., Pessin G. Exploiting the use of recurrent neural networks for driver behavior profiling. Proceedings of the 2017 International Joint Conference on Neural Networks (IJCNN).

[40] Guo F., Fang Y. (2013). Individual driver risk assessment using naturalistic driving data. Accid. Anal. Prev..

[41] Antin J., Lee S., Hankey J., Dingus T. (2011). Design of the In-Vehicle Driving Behavior and Crash Risk Study: In Support of the SHRP 2 Naturalistic Driving Study.

[42] Bärgman J., Lisovskaja V., Victor T., Flannagan C., Dozza M. (2015). How does glance behavior influence crash and injury risk? A ‘what-if’ counterfactual simulation using crashes and near-crashes from SHRP2. Transp. Res. F.

